# Study of the Possibility of Recycling of Technogenic Hafnium during Electron Beam Refining

**DOI:** 10.3390/ma15238518

**Published:** 2022-11-29

**Authors:** Katia Vutova, Vladislava Stefanova, Martin Markov, Vania Vassileva

**Affiliations:** 1 Institute of Electronics, Bulgarian Academy of Sciences, 1784 Sofia, Bulgaria; 2 Department of Metallurgy of Non-Ferrous Metals and Semiconductors Technologies, University of Chemical Technology and Metallurgy, 1756 Sofia, Bulgaria

**Keywords:** technogenic hafnium, recycling, electron beam melting, thermodynamic analysis, degree of removal

## Abstract

The possibility of removing metallic (such as Zr, Fe, Cr, and Zn) impurities and non-metallic (such as [O] and C) impurities from technogenic hafnium through single and double refining in the conditions of electron beam melting (EBM) has been studied. The influence of thermodynamic and kinetic parameters on the degree of removal of these impurities from the base metal under vacuum conditions and within a temperature interval of 2500 K to 3100 K is defined. The relative volatility of metal impurities and the stability of the oxides and carbides present in the base metal are evaluated. The possibility for complete removal of Fe, Cr, Zn, [O], and C during EBM is shown. In the case of double refining, at a temperature of 2700 K for 20 min, the maximum degree of removal of Zr is 46.8%, the achieved highest hafnium purity is 99.004%, and the overall effectiveness of the refining of hafnium from impurities is 53%. There is a correlation between the degree of removal of Zr and the micro-hardness of the Hf ingots obtained after EBM. The weight losses vary in the ranges of 1.5–5.8% and 1–8% under the studied single and double refining processes, respectively.

## 1. Introduction

The hafnium content in the Earth’s crust is 5.3 ppm (in weight). In nature, it is only found in combination with zirconium and thorium. The hafnium content in the Hafnon (Hf,Zr)SiO_4_ and Alwit (H,Th,Zr)SiO_4_∙xH_2_O zirconium minerals reaches up to 5%. However, its production is too limited—no more than 70 t per year, with most of the commercial hafnium being produced by zirconium refining. The separation of these two metals during the main production process is extremely complicated due to the great similarity in their chemical properties [1,2].

The specific requirements and restrictions on the zirconium content in pure hafnium require a complex approach and special additional chemical processing through liquid extraction, ion exchange, or fractional distillation of appropriate compounds [3,4,5]. This hampers and increases the costs for the overall production cycle of pure hafnium thus making it not environmentally friendly. A thorough review of the previously known hydro- and pyro-metallurgical methods for separation of the two metals was completed in [6]. The pyrometallurgical methods are based on the redox characteristics of hafnium and zirconium chlorides, the volatility of their tetrachlorides, their electrochemical properties, and based on molten salt–metal equilibrium.

The main application of hafnium is in the nuclear energy industry, which is due to its unique ability to absorb neutrons. Hafnium, as well as its isotopes, obtained via its irradiation, are characterized by a high value of the transverse section of absorption of heat neutrons (~100 barns) [7]. Thus, the preservation of the physical efficiency of the elements (based on Hf) is ensured for their prolonged operation in reactors. Hafnium alloys have excellent resistance to oxidation and corrosion at high temperatures [3,4]. They are highly in demand and are the preferred materials for application in the nuclear industry for the production of nuclear-safe and corrosion-resistant equipment for the transportation and processing of spent nuclear fuel. They are also used in shipbuilding, space equipment, turbine construction, in the production of thermoelectronic converters, electric heaters, and cathodes for plasma cutting and welding in a neutral and oxidizing environment. They are an indispensable material for the production of corrosion-resistant chemical and petroleum equipment. 

According to the Critical Raw Materials Alliance (CRM Alliance) data [8], the EU added hafnium to the list of critical raw materials in 2017. The recycling coefficient is about 1%, but it is expected to increase to 4% in the coming years because of the increased use of both pure Hf and its alloys and compounds. The recycling of this waste is a good opportunity to provide additional raw materials for the production of pure Hf and its alloys worldwide, and especially for countries that do not have the necessary natural resources for its production.

Previous studies have shown that the electron beam (EB) method is suitable for recycling scrap from refractory metals and already used metals and alloys [9,10,11]. By combining a high temperature and vacuum medium for the processes, this method provides good conditions for refining, mobility in the selection of technological parameters, and the possibility to obtain different sized ingots. It is an environmentally friendly and wasteless method that provides a high degree of purification from gases and impurities. The resulting material is characterized both by its uniformity with regard to the chemical composition and by its homogeneous, defectless microstructure and improved mechanical properties [12,13,14,15].

The possibility for recycling technogenic hafnium (fillings obtained from machining of industrial hafnium products) and impurities’ removal under electron beam melting (EBM) conditions is examined. Based on a thermodynamic analysis and the experiments conducted, an analysis is carried out on the effectiveness of the EB refining of metal hafnium from impurities such as Zr, Fe, Cr, Zn, [O], and C. The micro-hardness of the ingots obtained after recycling is determined.

## 2. Materials and Methods

The experiments for hafnium technogenic material melting were conducted in the “Physical problems of the e-beam technologies” laboratory of the Institute of Electronics, Bulgarian Academy of Sciences, using the e-beam melting furnace ELIT-60 with a beam power of 60 kW. The ELIT-60 installation (Leybold GmbH, Cologne, Germany) is equipped with one electron gun with an accelerating voltage of 24 kV and a vacuum melting chamber. The refined molten material solidifies in a water-cooled copper crucible with a moving bottom [15] and the operation vacuum pressure is 1 × 10^−3^ Pa.

The elemental composition of the waste Hf prior to the EBM (determined by ICP-OES) is given in Table 1. It shows that the total content of impurities is high (2.13%), with the content of zirconium (1.868%) and iron (0.147%) being the highest. The presence of dissolved [O] and C in the studied material indicates the presence of oxides and carbides.

In the present study, single and double electron beam melting and refining (EBMR) of technogenic hafnium have been performed. In the single refinement, the experiments were carried out at the following beam powers (P_b_): 12 kW (T = 2500 K), 15 kW (T = 2700 K), 16 kW (T = 2800 K), and 17 kW (T = 2900 K). At the lowest operating temperature (2500 K), the refining time (τ) was 5 min, while at the higher melting temperatures, the refining duration was 5 min and 8 min.

Four tests (A, B, C, and D) have been carried out during the double refining of the technogenic hafnium. The conditions of the experiments are determined on the basis of the results from the single refining. Test A includes initial refining (I-A) at T = 2500 K and τ = 5 min and second refining (II-A) at T = 2500 K and τ = 15 min. In test B, the first (I-B) and second (II-B) refining are performed at the same temperature (T = 2700 K) and refining time (τ = 8 min). Test C was performed at T = 2700 K and different refining durations: the first (I-C) at τ = 5 min and the second (II-C) at τ = 20 min. Test D includes initial refining (I-D) at T = 2900 K and τ = 5 min and second refining (II-D) at T = 3100 K and τ = 10 min.

The temperature was determined by an optical pyrometer QP-31 using special correction filters. 

The chemical composition of the initial material and the samples after the e-beam melting was determined by ICP-OES, a CS analyzer, and an ONH analyzer. The baseline and final concentrations were controlled for the base metal Hf and for the impurities such as Zr, Fe, Cr, Zn, [O], and C.

## 3. Results and Discussion

### 3.1. Thermodynamic Analysis of Refining Processes during EBM

The thermodynamic analysis of the possible chemical interactions occurring during the refining of technogenic hafnium from metallic (Zr, Cr, Fe, and Zn) and non-metallic impurities ([O] and C) under e-beam melting conditions was carried out on the basis of the Gibbs free energy (ΔG). The calculations were performed using the professional program for thermochemical calculations, HSC Chemistry ver.7.1, module “Reaction Equation”, taking into account the physical state of the substance [16]. 

The thermodynamic characteristics of the relevant elements and substances such as enthalpy, entropy, and coefficients of specific heat capacity, necessary for the calculations, were taken from the program database. The indices (s), (l), and (g) mean that the substance is in a solid, liquid, or gaseous state, respectively during EBMR.

The calculations were made at temperatures of 2500 K, 2700 K, 2800 K, 2900 K, and 3100 K and an operating pressure in the vacuum chamber of 1 × 10^−3^ Pa and these parameters correspond to the actual processing conditions of melting and refining of technogenic hafnium in the electron beam installation ELIT-60.

The pressure in the vacuum chamber is constant and the main parameters that affect the removal of impurities are the temperature, the physical state of the impurities during refining [15], and the mass transport of molten or solid particles to the reaction surface [17].

**Figure 1:**
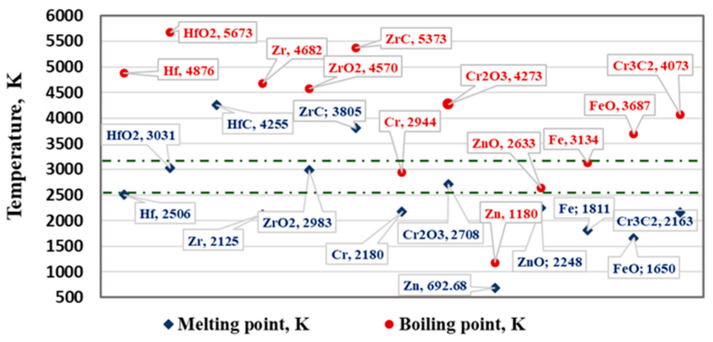
The melting and boiling temperatures of the studied metals and compounds.

The melting and boiling temperatures of the studied metals (Hf, Zr, Cr, Fe, and Zn), their oxides (such as HfO_2_, ZrO_2_, Cr_2_O_3_, FeO, and ZnO) and carbides (such as HfC, ZrC, and Cr_2_C_3_) are shown in Figure 1. The operating temperature range (2500–3100 K) of the EBMR process is marked in the figure by dashed lines.

It can be seen that in the entire investigated temperature range, the metallic impurities Zr, Fe, FeO, and Cr_3_C_2_ will be in a liquid state. At T = 2944 K, chromium changes from a liquid to a gaseous state. Chromium oxide (Cr_2_O_3_) changes into a liquid state at a temperature above 2700 K. At the highest operating temperature (3100 K), the oxides HfO_2_ and ZrO_2_ will also be in a molten state. Zinc is in a gaseous state within the entire operating temperature range, while ZnO changes from a liquid to a gaseous state at T = 2633 K. The melting temperatures of the hafnium and zirconium carbides are significantly higher—4255 K and 3805 K, respectively. These temperatures vary by about 30–40 K depending on the stoichiometric composition of the carbide [18]. Figure 1 shows the melting temperatures of stoichiometric carbides with respect to carbon (HfC and ZrC).

Therefore, under the conditions of EBM, the liquid metal is a multi-component system of liquid hafnium with metal impurities, their oxides, and carbides, which, depending on the operating temperature of the EBM process, will be in a solid, liquid, or gaseous state. 

The processes of e-beam melting and refining takes place mainly on the reaction surface of the liquid metal material (its interface with the vacuum) [15]. The vapor pressure of metal impurities and their compounds plays a crucial role in the refining process. The refining process can take place by degassing (when, for the partial pressure of a metal impurity *p_Me_*, the inequality (*p_Me_*) *>* (*p_Hf_*) is satisfied) and by distillation (evaporation of more volatile compounds of the metallic components) depending on the thermodynamic conditions (operating temperature and the pressure in the vacuum chamber). Sometimes non-volatile metal impurities form volatile components with high vapor pressure and are removed by distillation. 

For high efficiency of the refining process, the following inequalities should be satisfied:

(*p_MeO_*) > (*p_Me_*) > (*p_HfO2_*) > (*p_Hf_*) (1)

If the conditions (1) are not satisfied for the impurities, i.e., if (*p_HfO2_*) > (*p_MeO_*); (*p_HfO2_*) > (*p_Me_*) or (*p_HfO__2_*) < (*p_Hf_*), the losses of the base metal are higher and the refining process is not efficient. 

**Figure 2:**
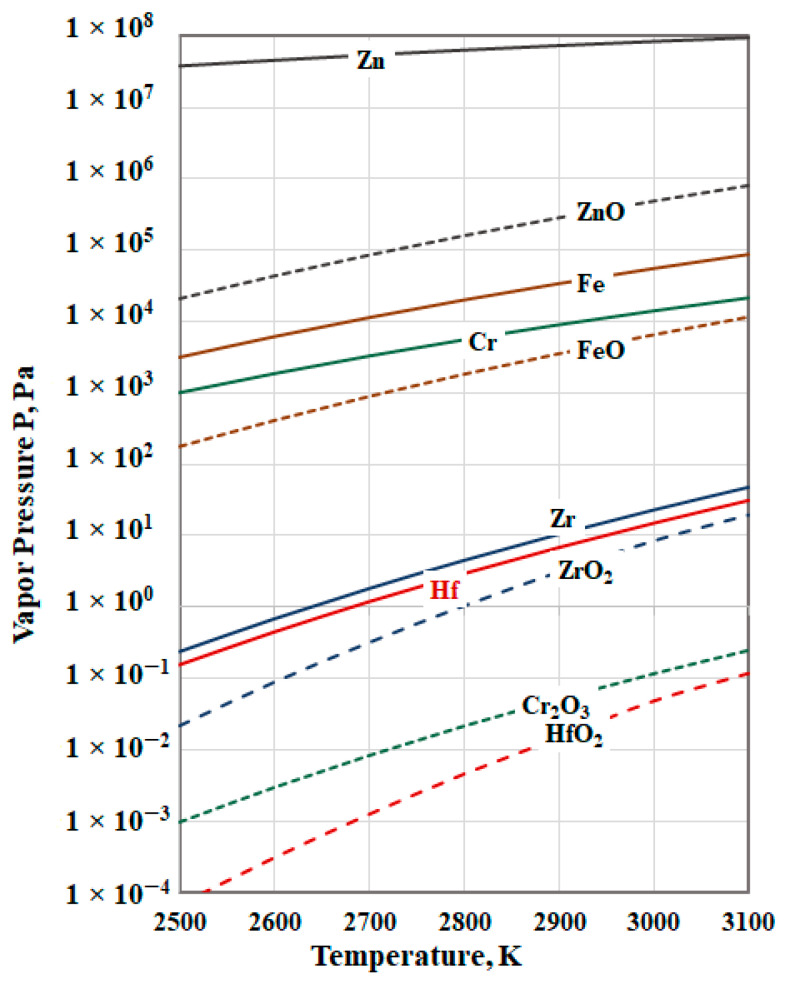
The vapor pressure of hafnium, metal impurities, and their oxides.

Figure 2 shows the calculated values of the vapor pressure of pure metals (Hf, Zr, Fe, Cr, and Zn) and their oxides (HfO_2_, ZrO_2_, FeO, Cr_2_O_3_, and ZnO). The vapor pressure of HfC is very low (10^−41^ Pa). Due to lack of thermodynamic data for ZrC and CrC, the vapor pressure of these two carbides is not calculated. 

The analysis of the obtained results shows that in the investigated temperature range, the vapor pressure of impurities such as Zn, Fe, and Cr and their oxides is significantly higher than that of hafnium. Since the condition (*p_Me_*) > (*p_Hf_*) is met for all three metals, they can be removed by evaporation. The vapor pressure of Zr is slightly higher than that of hafnium, indicating that the process is thermodynamically probable (*p_Zr_* > *p_Hf_*), but it will be accompanied by losses of the base metal. Figure 2 shows that with increasing the temperature, the dependence *p_ZrO_*_2_ = f_1_(T) approaches *p_Hf_* = f_2_(T). Hence, under certain conditions, ZrO_2_ can be removed by distillation. The vapor pressure of HfO_2_ and Cr_2_O_3_ is much lower than that of metallic hafnium, i.e., their removal by evaporation is thermodynamically impossible.

The efficiency of the refining process for multi-component metal systems can be evaluated by the relative volatility (*α*) [19], which is calculated by the equation:

(2) $$\alpha_{i}=\frac{p_{Hf}}{p_{i}}\frac{\sqrt{M_{i}}}{\sqrt{M_{Hf}}}$$

where *p_Hf_* and *p_i_* are the vapor pressures of hafnium and the metal impurity, respectively, and *M_Hf_* and *M_i_* are the molecular masses of hafnium and the metal impurity, respectively.

**Figure 3:**
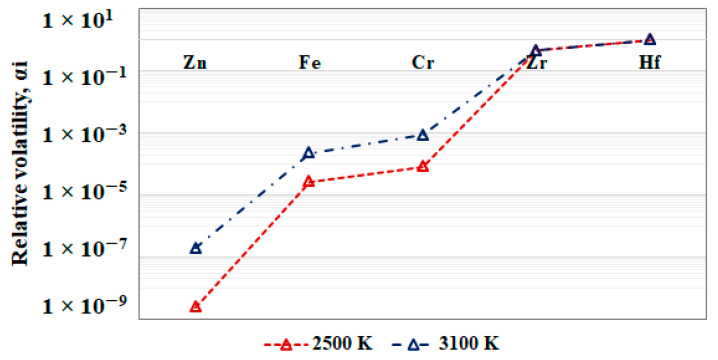
Values of the relative volatility *α_i_* for the metal impurities in hafnium at temperatures of 2500 K and 3100 K.

The values of the relative volatility *α_i_* of the Zr, Cr, Fe, and Zn impurities calculated by Equation (2) at operating temperatures of 2500 K and 3100 K are shown in Figure 3. 

The parameter *α_i_* varies in the range of 10^−9^–10^0^. It can be seen that for zinc, iron, and chromium *α_i_* << 1, i.e., these metals will be easily removed from hafnium. The value of the relative volatility of zirconium is close to 1 (*α_Zr_*~1). It is located to the left of hafnium and therefore the removal process is thermodynamically probable, but it will be accompanied by losses in the mass of hafnium. As the temperature rises, the relative volatility values of Zn, Fe, and Cr increase, while that of Zr is almost unchanged.

Thermodynamic evaluation of the possible chemical interactions in the system Hf-Me_i_-O is made on the basis of the following equations:

Hf(l) + 2[O] = HfO_2_(s,l) + ΔG_T_,_Hf/HfO2_, (3)

Me_i_(l,g) + [O] = Me_i_O(s,l,g) + ΔG_T_,_Mei/MeiO_, (4)

**Figure 4:**
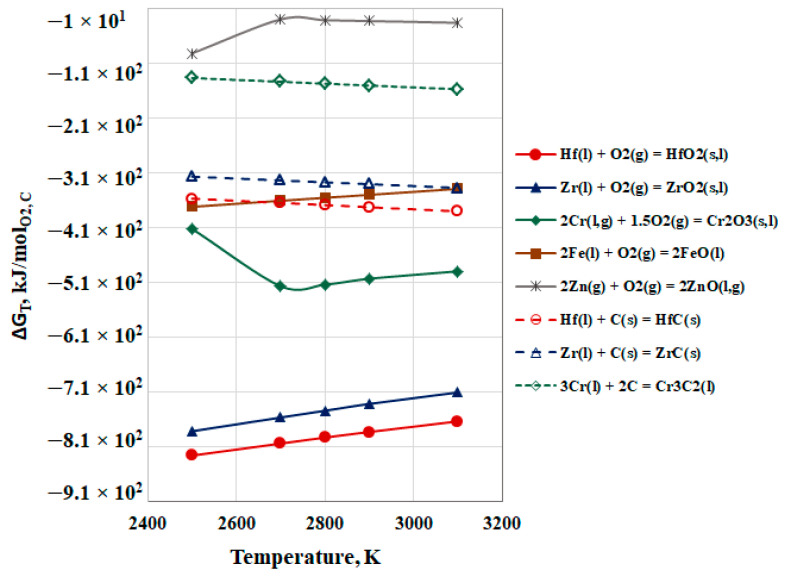
Influence of the temperature on ΔG_T_ of oxidation reactions of Hf(l) and Me_i_(s,l,g) under vacuum conditions.

HfO_2_(s,l) + 2Me_i_(l,g) = 2Me_i_O(s,l,g) + Hf(l) + ΔG_T_, (5)

where ΔG_T_,_Hf/HfO2_, ΔG_T_,_Mei/MeiO_, and ΔG_T_ are the Gibbs free energies. The calculations were completed at temperatures of 2500 K, 2700 K, 2800 K, 2900 K, and 3100 K and an operating pressure in the vacuum chamber of 1 × 10^−3^ Pa, taking into account the physical state of the metals and their oxides. The temperature dependences of the free energy of reactions of oxidation of hafnium (ΔG_T_,_Hf/HfO2_) and metallic impurities (ΔG_T_,_Mei/MeiO_) are shown in Figure 4.

It is clear that the liquid Hf(l) has the greatest affinity to oxygen. With an increase in temperature, ΔG_T_ increases from −826 kJ/mol_O2_ to −763 kJ/mol_O2_. Similar to hafnium, ΔG_T_ of oxidation of Zr(l) to ZrO_2_(s,l) also increases and approaches that of Hf (from −781 kJ/mol_O2_ to −710 kJ/mol_O2_). In both cases, during the transition of oxides from a solid to a liquid state, the change in free energy is ~−20 kJ/mol_O2_. 

Out of the other impurities present in liquid hafnium, the gaseous zinc is the least likely to oxidize to zinc oxide (from −91.5 kJ/mol_O2_ to −29.3 kJ/mol_O2_). A change in the slope of the curve at T = 2700 K is observed on the temperature dependence (ΔG_T_ = f(T), Figure 4), which is due to the transition of zinc oxide from a liquid to a gaseous state.

The change in the slope of the curve is also evident in the oxidation of chromium (Figure 4). After melting the Cr_2_O_3_, the Gibbs free energy of oxidation increases by ~100 kJ/mol_O2_, i.e., the oxidation process is thermodynamically more probable. As the temperature rises, the likelihood for an oxidation process slightly decreases. 

Within the overall temperature interval, the oxidation reaction Fe(l) to FeO(l) is thermodynamically probable. The influence of the temperature is insignificant, since the change of ΔG_T_ is ~40 kJ/mol_O2_ in the temperature interval of 2500 K to 3100 K.

Based on the dependencies obtained, it can be concluded that within the studied temperature range, the ability to form oxides decreases in the following direction HfO_2_(s,l) > ZrO_2_(s,l) > Cr_2_O_3_(s,l) > FeO(l) > ZnO(l,g).

In addition to dissolved oxygen, the material also contains carbon (Table 1). Figure 4 shows the temperature dependencies for the carbide formation reactions of hafnium, zirconium, and chromium. It can be seen that the probability for formation of HfC is the highest and the probability for formation of Cr_3_C_2_ is the smallest. The curves’ slope indicates that with increasing the temperature, the thermodynamic probability for their formation increases slightly. 

**Figure 5:**
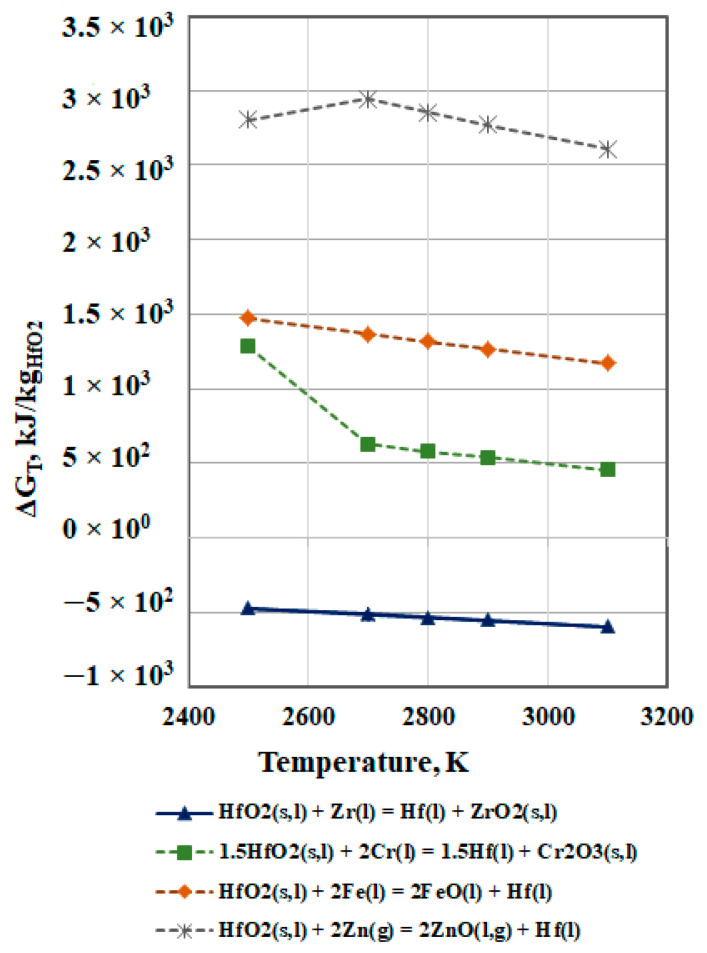
Free energy for interaction between HfO_2_ and metal impurities in vacuum.

The possibility for chemical reactions between HfO_2_(s,l) and metal impurities is described by Equation (5). The calculated values of the free energy ΔG_T_ in the studied temperature range are shown in Figure 5.

The analysis of the obtained dependences (ΔG_T_ = f(T)) in Figure 5 shows that in vacuum conditions, only the reaction HfO_2_(s,l) + Zr(l) = Hf(l) + ZrO_2_(s,l) is thermodynamically probable. As the temperature increases, the probability for this process slightly increases.

The positive values of the Gibbs energy of the chemical reactions between HfO_2_(s,l) and other impurities such as chromium, iron, and zinc indicate that they are thermodynamically impossible [20].

Hafnium and zirconium carbides form non-stoichiometric compounds with carbon that can dissolve even small amounts of oxygen [21]. The solubility of oxygen in ZrC is higher than that in HfC and depends on the stoichiometric composition of the carbide. For example, ZrC forms ZrC_1−x_O_x_ where x = 0.26 at 2123 K. HfC forms HfC_1−x_O_x_ where x = 0.1 at T = 2023 K. Further removal of oxygen and carbon is possible by releasing the gases CO(g) and CO_2_(g). The presence of these compounds was established during heating in graphite pots in the temperature range 2773–3073 K in an atmosphere of He [22]. 

In the current study, the thermodynamic assessment of the possibility for removing carbon and oxygen from technogenic hafnium is made on the basis of the following equations:

HfC(s) + Me_i_O(s,l,g) = HfO_2_(l) + Me_i_(l) + CO(g), (6)

HfC(s) + 2Me_i_O(s,l,g) = HfO_2_(l) + Me_i_(l) + CO_2_(g), (7)

ZrC(s) + Me_i_O(s,l,g) = ZrO_2_(l) + Me_i_(l) + CO(g), (8)

ZrC(s) + 2Me_i_O(s,l,g) = ZrO_2_(l) + Me_i_(l) + CO_2_(g) (9)

**Figure 6:**
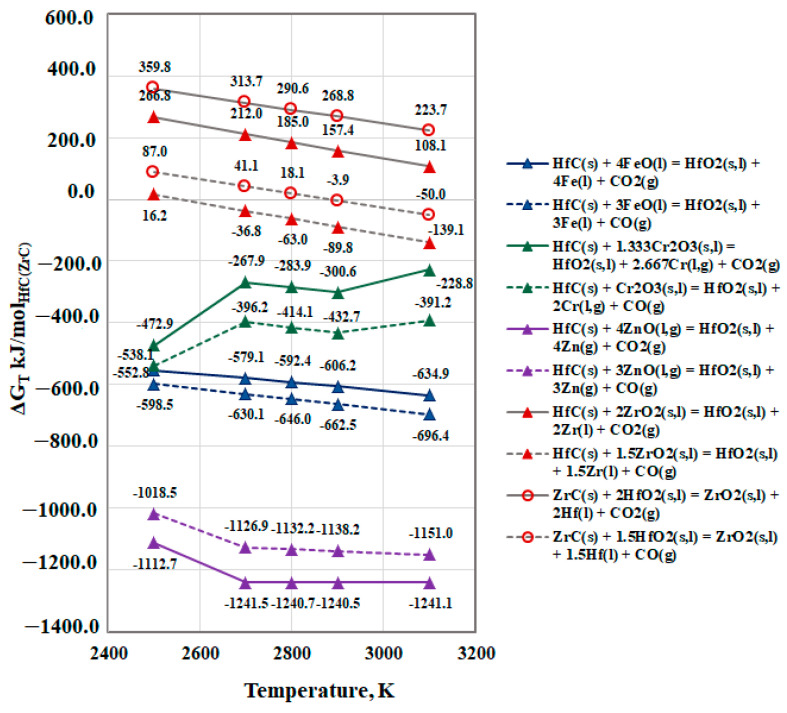
Free energy for interaction between HfC(s) and oxides of metal impurities in vacuum.

The results show that the chemical reactions of the two HfC and ZrC carbides with the oxides FeO, Cr_2_O_3_, and especially ZnO are thermodynamically possible. As the differences in the calculated free energy values for these chemical reactions of the two carbides are insignificant, only the results for HfC are shown in Figure 6 (Equations (6) and (7)). The chemical reactions resulting in obtaining CO_2_(g) and CO(g) are marked with solid and dashed lines, respectively. The typical twists in the curves are due to the change in the aggregate state of the substances. The same figure also presents the temperature dependencies of the Gibbs energy on the reactions of ZrC with HfO_2_(s,l) until CO(g) and CO_2_(g) are obtained. The positive values of the reactions for both carbides when obtaining CO_2_ indicate that they are thermodynamically impossible (Figure 6).

The calculated values of the Gibbs free energy of the HfC reactions with ZrO_2_ and of ZrC with HfO_2_ resulting in CO(g) show that the first reaction is thermodynamically probable at temperatures higher than 2700 K, while the second can only occur at the highest operating temperature (3100 K). Figure 6 indicates that CO_2_(g) and CO(g) will be present in the system within the studied temperature range under vacuum conditions. 

Based on the thermodynamic analysis above, it can be concluded that metal impurities (Zn, Fe, and Cr) will be removed mainly via evaporation. The high vapor pressure of FeO and ZnO indicates that they can also be removed via distillation. The removal of zirconium by evaporation is thermodynamically probable, but since the vapor pressure of zirconium is insignificantly higher than that of hafnium, the process will be accompanied by losses of the metal (Hf). Under vacuum conditions, at temperatures above 2500 K, only the reaction between HfO_2_ and Zr(l) is thermodynamically probable. The non-metallic impurities oxygen and carbon, present in technogenic hafnium, will most likely be removed in the form of CO(g) and CO_2_(g), which are products of the interaction between HfC and ZrC and the ZnO, FeO, and Cr_2_O_3_ oxides.

### 3.2. Refining Efficiency during EBMR of Technogenic Hafnium

Table 2 shows the chemical compositions of ingots obtained after a single refinement of used hafnium under different technological modes.

The analysis of the results shows that impurities such as Zn, Fe, and Cr are easily removed from the liquid hafnium. Zinc is completely removed, and the degree of removal of Fe and Cr is above 95% at the lowest operating temperature of 2500 K and a 5 min retention time. At higher operating temperatures, all three metals are completely removed. They are removed both by degassing and by distillation, since the vapor pressure of these metals and their oxides significantly exceeds that of hafnium (Figure 2).

**Figure 7:**
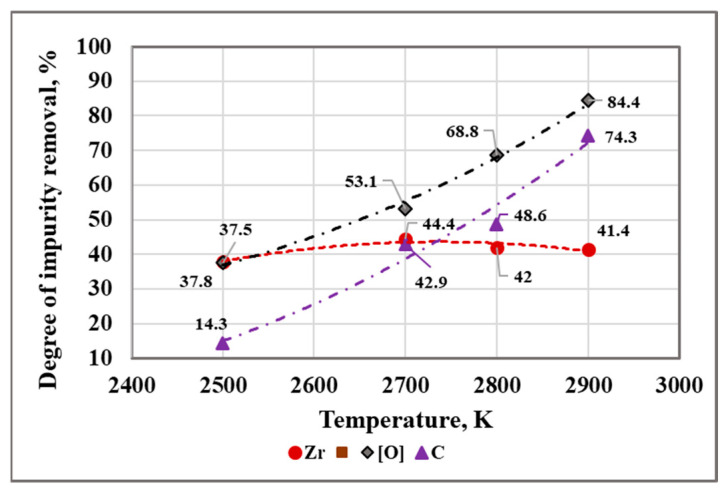
Influence of melting temperature on the degree of impurity removal from technogenic hafnium in vacuum for τ = 5 min.

**Figure 8:**
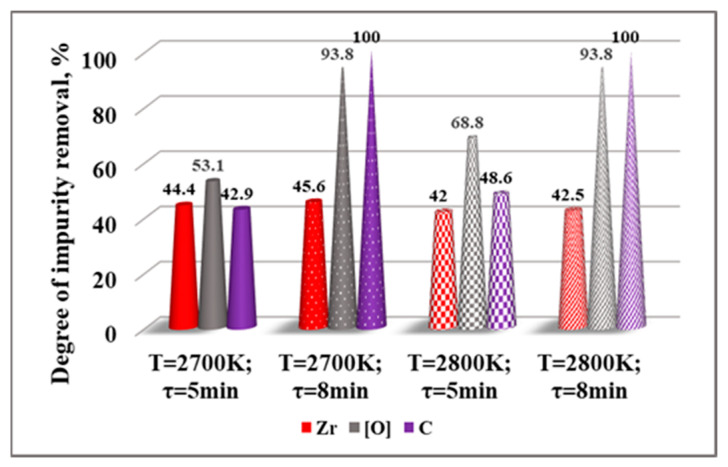
Influence of residence time on the degree of impurity removal at T = 2700 K and T = 2800 K from technogenic hafnium in vacuum.

In order to easily interpret the behavior of other impurities (Zr, [O], and C) during the EBM process, graphic dependencies were built for the impact of the temperature during the refining time 5 min (Figure 7) and the refining time at T = 2700 K and T = 2800 K (Figure 8) on the degree of their removal (*β_i_*) from liquid hafnium. The removal rate values (*β_i_*) are calculated from:

(10) $$\beta_{i}=\frac{C_{i\left(initial\right)}-C_{i\left(final\right)}}{C_{i\left(initial\right)}}100\%$$

where $C_{i\left(initial\right)}$ and $C_{i\left(final\right)}$ are the initial and final concentrations of the *i*-th element in the material, respectively.

The analysis of the results obtained indicates that the maximum degree of removal of Zr (45.6%) and the best purification of hafnium technogenic material (98.982%) is achieved at 2700 K and a refining time of 8 min with a weight loss of 5.1%. With an increase in temperature to 2900 K and τ = 5 min, the degree of refining of Zr decreases to 41.4%. The influence of the refining time on *β_Zr_* is negligible—about 1–2%.

Figure 7 and Figure 8 show that both parameters, temperature and refining time, significantly influence the degree of removal of oxygen and carbon. When the refining time is 5 min and the temperature increases from 2500 K to 2900 K, the degree of removal of oxygen increases from 37.5% to 84.4% and that of carbon increases from 14.3% to 74.3%. If the refining time is extended to 8 min at T = 2800 K, the degree of removal of oxygen reaches up to 93.8% and carbon is completely removed. This can be explained by possible chemical reactions of HfC and ZrC carbides with zinc, iron, and chromium oxides, which result in gaseous CO(g) and CO_2_(g) products

**Figure 9:**
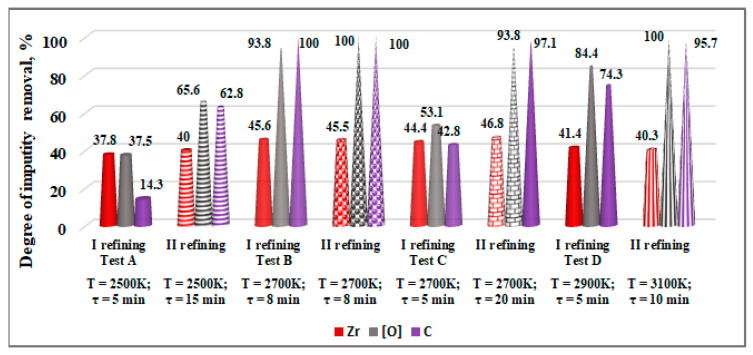
Influence of melting temperature and time on the degree of impurity removal at single and double refining from technogenic hafnium in vacuum.

The results obtained show that the maximum degree of removing Zr is 45.6% and the overall effectiveness of refining technogenic hafnium does not exceed 53%. Therefore, some ingots from the first refining were refined a second time. The conditions for the first and second refining and the composition of the resulting ingots are given in Table 3, and the degree of removal of Zr, [O], and C are given in Figure 9.

The analysis of the results shows that when refining technogenic hafnium for a second time at a temperature of 2500 K and an extended time of up to 15 min, the content of zirconium slightly decreases (from 1.162% to 1.12%). The degree of removal of Zr increases by ~2.2%, while those of [O] and C increase significantly, up to 65.6% and 62.8%, respectively.

The lowest zirconium content (0.994%) in hafnium and the highest hafnium purity of 99.004% is achieved in test C (T = 2700 K, τ = 20 min) with a weight loss of 8%. The maximum degree of Zr removal is 46.8% and it is slightly higher than the result obtained during single refining (45.6%). Unlike zirconium, the degree of removal of [O] and C is more than 90%.

In test D (with the highest operating temperature and double refining), the degree of removal of Zr decreases to 40.3%. The overall effectiveness of Hf refining is also reduced (47.4%).

Based on the thermodynamic and experimental studies, a hypothesis is proposed to reduce the degree of removal of Zr and the complete removal of [O] and C at high operating temperatures. The thermodynamic analysis performed has shown that the following interactions can take place under temperatures above 2900 K under vacuum conditions:

HfO_2_(l) + Zr(l) = Hf(l) +ZrO_2_(l), (11)

HfC(s) + 1.5ZrO_2_(l) = HfO_2_(l) + 1.5Zr(l) + CO(g), (12)

The result of the interactions according to reactions (11) and (12) is the reaction:

HfC(s) + 0.5ZrO_2_(l) = Hf(l) +0.5Zr(l) + CO(g), (13)

It can be seen that the end products from the chemical reactions taking place in the Hf-Zr-O-C system will be liquid hafnium and zirconium and a gaseous component CO(g).

The calculated value of the Gibbs free energy of the reaction (13) at a temperature of 3100 K and a pressure in the vacuum chamber of 10^−3^ Pa is −946 kJ/kg_Zr_. It is negative and commensurate with ΔG_T_ of the other reactions (11) and (12) (−1372 kJ/kg_Zr_ and −1667 kJ/kg_Zr_). Removal of the gas stream (CO(g)) will facilitate the reaction in the direction of obtaining Hf(l) and Zr(l).

The results obtained so far completely confirm the conclusions made on the basis of the thermodynamic analysis of the behavior of the impurities present in technogenic hafnium. The low degree of removal of zirconium by evaporation in EBM conditions is due to the small differences in the vapor pressure of zirconium and hafnium within the studied temperature intervals and the ability to form Zr(l) during the reaction in the Hf-Zr-O-C system.

The average values of micro-hardness measured according to Vickers (μHV) and the standard deviations at a load of 0.2 kgf/mm^2^ of the hafnium specimens obtained after single and double refining are presented in Table 4 and Table 5, respectively.

It can be seen that the removal of impurities during EBM leads to homogenization of the sample and the reduction of its micro-hardness. The analysis of the results shows that after single refining, the lowest micro-hardness of the ingot (2378 MPa) is obtained at T = 2700 K and τ = 8 min. With double refining of hafnium at the same temperature and a longer refining time (20 min), the measured micro-hardness of the ingot decreases slightly (2321 MPa). With the further increase in temperature to 2900 K in single refining, the micro-hardness of the sample increases by ~9%, and with double refining and a higher temperature T = 3100 K, it increases by ~6%. The measured values of the specimens’ micro-hardness are in accordance with the degree of removal of impurities and the chemical composition of the samples after single and double refining.

## 4. Conclusions

The possibility for removing metallic (Zr, Cr, Fe, and Zn) impurities and non-metallic ([O] and C) impurities from technogenic hafnium during electron beam melting has been examined in the paper. Based on the thermodynamic analysis and experimental results from single and double refining in different technological modes, the following conclusions can be made:

- In the temperature range of 2500–3100 K in vacuum conditions, the evaporation of the impurities Zn, Fe, and Cr, whose vapor pressure is significantly greater than that of hafnium, is most likely. The Zr vapor pressure is slightly higher, but close in value to that of Hf and its evaporation is thermodynamically probable, but it will be accompanied by losses of the base metal.
- The non-metallic impurities, oxygen and carbon, present in technogenic hafnium will most likely be removed in the form of CO(g) and CO_2_(g), which are products from the chemical reaction between HfC and ZrC and ZnO, FeO and Cr_2_O_3_ oxides.
- It has been found that with single refining, the maximum degree of removal of Zr (45.6%) and the best purification of hafnium technogenic material (98.982%) is achieved at a temperature of 2700 K and a refining time of 8 min. With the increase in the temperature to 2900 K, the degree of removal of Zr decreases to 41.4%, which is due to both the very small difference in the vapor pressure between Zr and Hf as well as being due to the possible chemical reactions in the Hf-Zr-O-C system, which result in liquid zirconium.
- The results from the double refining of hafnium indicate that the degree of removal of zirconium slightly increases even with prolonged refining. The lowest content of zirconium in the tested material and hafnium with the highest purity of 99.004% is achieved at T = 2700 K for 20 min and the effectiveness of refining of technogenic hafnium is 53%.
- The lowest value of hafnium micro-hardness is obtained at the highest degree of zirconium removal.

The results obtained confirm the significant similarity in the physical and chemical properties of the two metals (Hf and Zr), which hampers separation. The study showed that zinc, iron, chromium, oxygen, and carbon can be completely removed under the EBM of technogenic hafnium, and the maximum level of zirconium removal is 46.8%.

## Figures and Tables

**Table 1:** Chemical composition of technogenic hafnium before being treated by electron beam melting and refining (in mass. %).

C_Hf_	C_Zr_	C_Fe_	C_Cr_	C_Zn_	C_[O]_	C_C_
97.870	1.868	0.147	0.042	0.006	0.032	0.035

**Table 2:** Process parameters and chemical composition of ingots before and after single refining of technogenic hafnium during EBM.

Probe	T, K	Time min	Concentration of Elements in Mass. %							Σ_imp_, %
			Hf	Zr	Fe	Cr	Zn	[O]	C	
Hf-00	Initial probe		97.87	1.868	0.147	0.042	0.006	0.032	0.035	2.13
Hf-03	2500	5	98.78	1.162	0.006	0.002	<0.001	0.02	0.03	1.220
Hf-01	2700	5	98.925	1.039	0.001	<0.001	<0.001	0.015	0.02	1.075
Hf-08		8	98.982	1.016	<0.001	<0.001	<0.001	0.002	<0.001	1.018
Hf-10	2800	5	98.889	1.083	<0.001	<0.001	<0.001	0.01	0.018	1.111
Hf-09		8	98.925	1.073	<0.001	<0.001	<0.001	0.002	<0.001	1.075
Hf-05	2900	5	98.891	1.095	<0.001	<0.001	<0.001	0.005	0.009	1.109
Hf-04		8	98.892	1.105	<0.001	<0.001	<0.001	0.003	<0.001	1.108

**Table 3:** Process parameters and chemical composition of ingots before EBM and after double e-beam refining of technogenic hafnium.

Test	Refining	T	τ	Concentration of Elements in Mass. %							Σ_imp_,%
		K	Min	Hf	Zr	Fe	Cr	Zn	[O]	C	
		Initial probe		97.87	1.868	0.147	0.042	0.006	0.032	0.035	2.13
A	A-I	2500	5	98.78	1.162	0.006	0.002	<0.001	0.02	0.03	1.220
	A-II	2500	15	98.827	1.120	<0.001	<0.001	<0.001	0.011	0.013	1.173
B	B-I	2700	8	98.982	1.016	<0.001	<0.001	<0.001	0.002	<0.001	1.018
	B-II	2700	8	98.982	1.018	<0.001	<0.001	<0.001	<0.001	<0.001	1.018
C	C-I	2700	5	98.925	1.039	0.001	<0.001	<0.001	0.015	0.02	1.075
	C-II	2700	20	99.004	0.994	<0.001	<0.001	<0.001	0.002	0.001	0.996
D	D-I	2900	5	98.891	1.095	<0.001	<0.001	<0.001	0.005	0.009	1.109
	D-II	3100	10	98.88	1.115	<0.001	<0.001	<0.001	<0.001	0.005	1.120

**Table 4:** Average micro-hardness and standard deviation measured for hafnium specimens produced under different technological modes after single EBMR.

Probe	Process Parameters			Hardness μHV	Standard Deviation
	T, K	τ, Min		MPa	
Hf-00	Initial probe			3166	+/−169
Hf-03	2500		5	2802	+/−149
Hf-01	2700		5	2620	+/−54
Hf-08			8	2378	+/−93
Hf-10	2800		5	2624	+/−94
Hf-09			8	2568	+/−50
Hf-05	2900		5	2612	+/−57
Hf-04			8	2593	+/−76

**Table 5:** Average micro-hardness and standard deviation measured for hafnium specimens produced after double e-beam melting and refining.

Test	Refining (Probe No)	Process Parameters		Hardness μHV	Standard Deviation
		T, K	τ, Min	MPa	
A	I (Hf-03)	2500	5	2802	+/−149
	II (Hf-06-04-3)	2500	15	2742	+/−94
B	I (Hf-08)	2700	8	2378	+/−93
	II (Hf-073)	2700	8	2357	+/−65
C	I (Hf-01)	2700	5	2620	+/−54
	II (Hf-01-10-3)	2700	20	2321	+/−52
D	I (Hf-05)	2900	5	2612	+/−57
	II (Hf-05-02-3)	3100	10	2697	+/−88
